# Do Ateroma ao Índice Aterogênico, Evidências Seculares

**DOI:** 10.36660/abc.20230819

**Published:** 2024-02-16

**Affiliations:** 1 Hospital Angelina Caron Campina Grande do Sul PR Brasil Hospital Angelina Caron, Campina Grande do Sul, PR – Brasil; 2 Universidade Federal do Paraná Faculdade de Medicina Curitiba PR Brasil Universidade Federal do Paraná Faculdade de Medicina, Curitiba, PR – Brasil

**Keywords:** Aterosclerose/fisiopatologia, Ateroma, Placa Aterosclerótica, Tomografia Computadorizada/métodos, Lesões do Sistema Vascular, Coenzima A, Fatores de Risco

A descoberta da aterosclerose foi descrita pela primeira vez pelo paleontólogo austríaco Johan N. Nepomuk Czemark quando observou grandes placas calcificadas na artéria torácica ascendente em múmias.^1^ Outros colegas investigadores tiveram descobertas semelhantes, mas um caso específico ganhou atenção. Murphy et al., em 2003, usando tomografia computadorizada, revelaram em uma múmia de 5.300 anos placas ateroscleróticas na aorta, coronárias, carótidas e artérias ilíacas.^2^

No início de 1900, foi publicado um número significativo de estudos sobre aterosclerose. Destaque para Alexander I. Ignatowski, que publicou em 1908 um estudo pioneiro associando alimentos com colesterol total (CT) elevado ao aumento da aterosclerose em modelo animal.^3^ Em 1910, Adolf Windaus, que ganharia o prêmio Nobel de química em 1928, demonstrou que as placas ateroscleróticas tinham 25 vezes mais colesterol do que uma parede arterial normal.^4^

Em 1912, Nikolau N. Anitschkow e Semen Chalatov replicaram o trabalho de Ignatowski, demonstrando que em coelhos alimentados com uma dieta rica em colesterol purificado, uma maior incidência de lesões vasculares estava associada a níveis elevados de colesterol plasmático. Além disso, estabeleceram a presença de elementos celulares, incluindo macrófagos, linfócitos e células lisas.^5^

Na época, em 1904, numerosos autores estudaram as bases fisiopatológicas da aterosclerose, termo introduzido pelo patologista Felix Marchand, que indicava o conteúdo lipídico nas lesões arteriais.^6^ Após essa descoberta, surgiram duas teorias opostas tentando explicar a inflamação base da placa aterosclerótica, uma de Rudolf Virchow e outra de Carl von Rokitansky.^7,8^

Outra descoberta inovadora foi feita por Rudolph Schoenheimer em 1933, quando comprovou que os animais podiam sintetizar colesterol e que essa síntese poderia ser inibida, trazendo à tona o conceito que levou à descoberta do receptor de lipoproteína de baixa densidade (LDL-R).^9^

Na década de 50, foram descobertos aspectos biológicos da ligação entre a molécula de colesterol e a Coenzima A, chamando a atenção para o Prêmio Nobel de Fisiologia de 1964 para Konrad Block e Feodor Lynen.^10^

A partir dessas descobertas, a "Era do Colesterol" terminou, dando espaço à "Era LDL-C". Essa fase foi resultado da pesquisa de John Gofman em 1955 que determinou as lipoproteínas pela sua densidade.^11^

A partir de 1973, Goldstein e Brown publicaram uma série de artigos sobre a atividade da enzima HMG CoA redutase, o LDL-c e seus receptores. Esses estudos pioneiros foram premiados em 1986 com o prêmio Nobel.^12,13^ Naquela época, Akira Endo já havia conhecido as estatinas do fungo do arroz, sendo esta a primeira substância comercializada em 1976.^14^

Ao longo do século, com todo esse conhecimento acumulado, surgiram estudos epidemiológicos para estabelecer os fatores de risco em escala populacional para a aterosclerose. O estudo principal foi a coorte de Framingham iniciada após a Segunda Guerra Mundial. Até hoje é uma das coortes que gerou maior número de artigos sobre o assunto. A maioria dos conceitos utilizados hoje na prevenção de doenças cardiovasculares foram determinados a partir do estudo de Framingham.

O parâmetro de referência para os índices aterogênicos foi a razão entre o colesterol total (CT) e a lipoproteína de alta densidade (HDL-C), denominado índice de Castelli (CT/HDL-C), em 1983.^15^ Esse índice também estabelece a relação entre o LDL -C e HDL-C (LDL-C/HDL-C). O objetivo deles era prever o risco cardiovascular proposto por Millán e Pintó.^16,17^

Outro índice importante foi o de triglicerídeos e HDL (TG/HDL), proposto por Gaziano. O índice TG/HDL elevado foi associado a maior risco de infarto do miocárdio (IM) em pacientes menores de 76 anos internados por IM e sem doença coronariana prévia.^18^ Luz e colaboradores demonstraram em 374 pacientes aumento progressivo do risco de doença CV, avaliados pelo índice de Friesinger, com maiores quartis da relação TG/HDL.^19^

Associados aos índices ateroscleróticos, temos hoje alguns métodos prognósticos aprimorados, como por exemplo, a perfusão periférica que utiliza oximetria de pulso para avaliar a disfunção endotelial, demonstrada por Menezes e colaboradores.^20^

O estudo de Araújo et al.^21^ utilizou diferentes índices ateroscleróticos, Castelli I (IC-I, CT/HDL-C), Castelli II (IC-II, LDL-C/HDL-C), índice combinado de lipoproteínas (CLI)(CTxTGxLDL/HDL), o índice aterogênico plasmático índice (PAI) obtido a partir do log10(TG/HDL), e índice de perfusão periférica no intervalo de 90-12 segundos (ΔIPP90-120) para avaliar seu valor como preditores de aterosclerose clínica. No estudo evidenciou-se que o PAI e o ΔIPP90-120 foram preditores superiores em todos os modelos de regressão logística quando o ponto de corte foi >0,06 e ≤56,6, respectivamente. A associação foi consistente entre os três modelos, com diminuição do tamanho do efeito à medida que os ajustes foram mais refinados. Ambos os índices apresentaram AUC aceitável, em torno de 80%, com grande valor preditivo negativo, em torno de 85% para ambos.

No último século, observaram-se fases importantes do conhecimento sobre CT e LDL-C com evidências de alta qualidade desses biomarcadores na predição do risco CV. O estudo de Araújo et al.^21^ acrescentou mais evidências relacionadas aos índices aterogênicos que refinam a estratificação de risco, aumentando a capacidade dos médicos de tratar os pacientes de maneira individualizada.

Embora existam evidências robustas sobre biomarcadores para a previsão de resultados cardiovasculares, os médicos ainda precisam desmistificar postagens nas redes sociais, artigos que não são revisados por pares e outras fontes que tentam antagonizar as evidências atuais sem o devido rigor científico, tornando mais difícil pôr em prática a medicina baseada em evidências.
