# Supplementary material for: Do Ateroma ao Índice Aterogênico, Evidências Seculares
Source: Arq Bras Cardiol. 2024 Feb 16;120(12):e20230819. [Article in Portuguese] doi: 10.36660/abc.20230819 (PMC11021034; doi:10.36660/abc.20230819)
Supplement: Translation

# Translation: From Atheroma to Atherogenic Index, Secular Evidence

**Affiliations:** 1 Campina Grande do Sul PR Brazil Hospital Angelina Caron, Campina Grande do Sul, PR – Brazil; 2 Curitiba PR Brazil Universidade Federal do Paraná Faculdade de Medicina, Curitiba, PR – Brazil

**Keywords:** Atherosclerosis/physiopathology, Atheroma, Plaque, Atherosclerosis, Tomography, X-Ray Cmputed/methods, Vascular System Injuries, Coenzyme A, Risk Factors

The discovery of atherosclerosis was first described by the Austrian paleontologist Johan N. Nepomuk Czemark when he observed great calcified plaques in the ascending thoracic artery in mummies.^1^ Other fellow researchers had similar findings, but one particular case gained attention. Murphy et al., in 2003, using computer tomography, revealed in a 5300-year-old mummy atherosclerotic plaques in the aorta, coronaries, carotids, and iliac arteries.^2^

In early 1900, a significant number of studies about atherosclerosis were published. Highlight for Alexander I. Ignatowski, who published in 1908 a pioneer study associating high total cholesterol (TC) foods with enhanced atherosclerosis in an animal model.^3^ In 1910, Adolf Windaus, who would win a Nobel prize in chemistry in 1928, demonstrated that the atherosclerotic plaques had 25 times more cholesterol than a normal artery wall.^4^

In 1912, Nikolau N. Anitschkow and Semen Chalatov replicated Ignatowski's work, demonstrating that in rabbits fed with a high purified cholesterol diet, a higher incidence of vascular injuries was associated with high levels of plasma cholesterol. Beyond that, they established the presence of cellular elements, including macrophages, lymphocytes, and smooth cells.^5^

At the time, in 1904, numerous authors studied the pathophysiology basis of atherosclerosis, a term that was introduced by the pathologist Felix Marchand, who indicated the lipid content in arterial injuries.^6^ Following this discovery, two opposite theories surged trying to explain the inflammatory basis of the atherosclerotic plaque, one from Rudolf Virchow and the other from Carl von Rokitansky.^7,8^

Another groundbreaking discovery was made by Rudolph Schoenheimer in 1933, when he proved that animals could synthesize cholesterol and that this synthesis could be inhibited, bringing up the concept that led to the discovery of the low-density lipoprotein receptor (LDL-R).^9^

In the fifties, biological aspects of the ligation between the cholesterol molecule and Coenzyme A were discovered, calling attention to the Nobel Prize award in Physiology in 1964 for Konrad Block and Feodor Lynen.^10^

Since these discoveries, the "Cholesterol Era" came to an end, giving space to the "LDL-C Era". This phase was the result of John Gofman's research in 1955 that determined the lipoproteins by their density.^11^

Beginning in 1973, Goldstein and Brown published a series of articles about the HMG CoA reductase enzyme activity, the LDL-c, and receptors. Over these pioneering studies, they were awarded in 1986 with a Nobel prize.^12,13^ At that time, Akira Endo had already learned about the statins from the rice fungus, being this the first substance commercialized in 1976.^14^

Over the century with all this knowledge accumulated, epidemiological studies surged to establish the risk factors on a populational scale for atherosclerosis. The main study was the Framingham cohort that started after the Second World War. Until today is one of the cohorts that generated the highest number of articles about the subject. Most of the concepts used today in the prevention of cardiovascular diseases were determined from the Framingham study.

The benchmark for the atherogenic indexes was the ratio between the total cholesterol (TC) and high-density lipoprotein (HDL-C), called the Castelli index (TC/HDL-C), in 1983.^15^ This index also sets up the relation between LDL-C and HDL-C (LDL-C/HDL-C). The purpose of them was to predict the cardiovascular risk as proposed by Millán and Pintó.^16,17^

Another important index was the triglycerides and HDL (TG/HDL), proposed by Gaziano. The high TG/HDL index was associated with a higher risk of myocardial infarction (MI) in patients under 76 years old admitted by MI and without previous coronary disease.^18^ Luz and collaborators demonstrated in 374 patients a progressive risk increase in CV disease, evaluated by the Friesinger index, with higher quartiles of TG/HDL ratio.^19^

Associated with the atherosclerotic indexes, today we have some enhanced prognostic methods, for example, the peripheral perfusion that uses pulse oximetry to evaluate endothelial dysfunction, demonstrated by Menezes and collaborators.^20^

The study by Araújo et al.^21^ used different atherosclerotic indexes, Castelli I (IC-I, TC/HDL-C), Castelli II (IC-II, LDL-C/HDL-C), combined lipoprotein index (CLI)(CTxTGxLDL/HDL), the plasmatic atherogenic index (PAI) obtained from log10(TG/HDL), and the peripheral perfusion index in the 90-12 seconds interval (ΔIPP90-120) to evaluate their value as predictors of clinical atherosclerosis. In the study, it was evidenced that the PAI and ΔIPP90-120 were superior predictors in all logistic regression models when the cutoff was >0,06 and ≤56.6, respectively. The association was consistent within the three models, with a decrease in the effect size as the adjustments were more refined. Both indexes had an acceptable AUC, around 80%, with a great negative predictive value, roughly 85% for both.

In the last century, it was observed important phases of the knowledge regarding TC and LDL-C with high-quality evidence of these biomarkers in predicting CV risk. The study from Araújo et al.^21^ added more evidence related to atherogenic indexes that refine risk stratification, enhancing the ability of clinicians to treat patients in an individualized manner.

Even though robust evidence about biomarkers for the prediction of CV outcomes is available, clinicians still have to demystify posts on social media, articles that are not peer-reviewed, and other sources that try to antagonize with the current evidence without proper scientific rigor, making it harder to put in practice evidence-based medicine.
